# Correction: Bienfait et al. Evaluation of 8% Capsaicin Patches in Chemotherapy-Induced Peripheral Neuropathy: A Retrospective Study in a Comprehensive Cancer Center. *Cancers* 2023, *15*, 349

**DOI:** 10.3390/cancers15143613

**Published:** 2023-07-14

**Authors:** Florent Bienfait, Arthur Julienne, Sabrina Jubier-Hamon, Valerie Seegers, Thierry Delorme, Virginie Jaoul, Yves-Marie Pluchon, Nathalie Lebrec, Denis Dupoiron

**Affiliations:** 1 Anaesthesiology and Pain Department, Institut de Cancérologie de l’Ouest, 49100 Angers, France; 2 Biometrics Department, Institut de Cancérologie de l’Ouest, 49100 Angers, France; 3 Pain Management Consultation Center, Centre Hospitalier Départemental Vendée, 85925 La Roche-sur-Yon, France

In the original publication [1], there was a mistake in the published Figure 6. The labels of the two lines in Figure 6 should be reversed. “Three or more applications” should be above “Two applications or fewer”. The corrected Figure 6 appears below.

The authors state that the scientific conclusions are unaffected. This correction was approved by the Academic Editor. The original publication has also been updated.

## Figures and Tables

**Figure 6:**
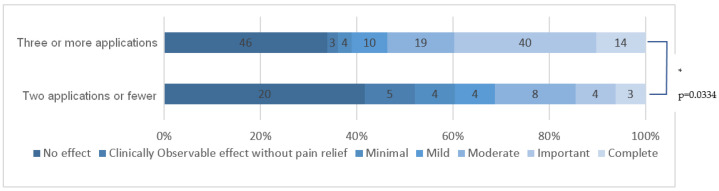
HCCP efficacy for patients who received three or more applications compared to those who received fewer. * means that there is a statistically significant difference.
